# Iatrogenic Gastroesophageal Junction Perforation During Emergency Endoscopic Retrograde Cholangiopancreatography in a 91-Year-Old Woman with Choledocholithiasis and Cholangitis

**DOI:** 10.1055/a-2877-2006

**Published:** 2026-06-03

**Authors:** Songming Ding, Yangjun Gu, Yang Liu, Shusen Zheng, Qiyong Li

**Affiliations:** 1 Division of Hepatobiliary and Pancreatic Surgery 636046 Shulan (Hangzhou) Hospital Affiliated to Zhejiang Shuren University, Shulan International Medical CollegeHangzhouZhejiang ProvinceChina; 2 Endoscopy Center 636046 Shulan Hangzhou Hospital Affiliated to Zhejiang Shuren University Shulan International Medical CollegeHangzhouChina

Acute calculous cholangitis carries a high mortality rate in elderly patients and frequently necessitates urgent endoscopic retrograde cholangiopancreatography (ERCP 1​ ). Although rare, procedure-related gastroesophageal junction (GEJ) perforation is a life-threatening complication. 2​ We report a case of iatrogenic GEJ perforation occurring during emergency ERCP in a 91-year-old woman, which was successfully managed with immediate endoscopic clipping.

**Fig. 1:**
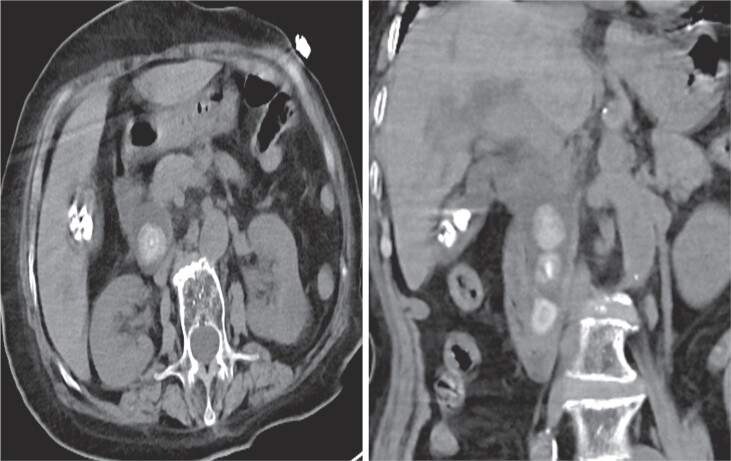
Admission whole-abdomen computed tomography (CT) showing multiple common bile duct (CBD) stones, intra- and extrahepatic biliary dilation, and acute calculous cholecystitis.

The patient presented with a 2-week history of abdominal pain and a 1-day history of fever. Physical examination revealed a temperature of 39.1°C and epigastric tenderness. Laboratory investigations indicated leukocytosis, elevated C-reactive protein, and hepatic dysfunction. Whole-abdomen computed tomography (CT) confirmed acute calculous cholangitis, demonstrating multiple common bile duct (CBD) stones, intra- and extrahepatic biliary dilation, and concomitant calculous cholecystitis ( Fig. 1 ).

**Fig. 2:**
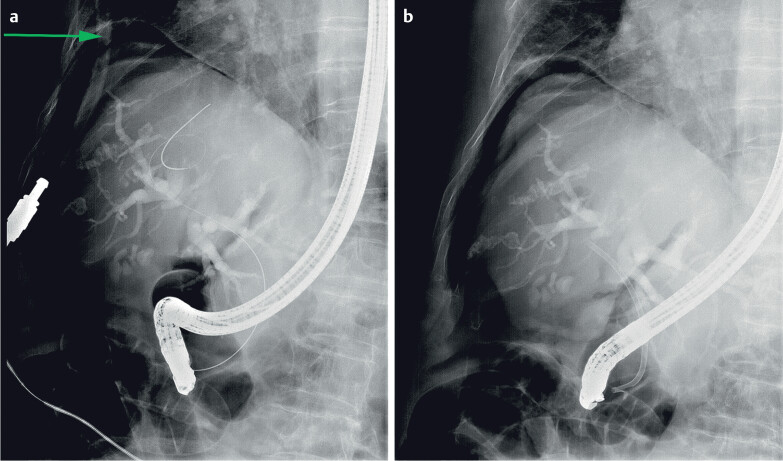
Intra-procedural endoscopic retrograde cholangiopancreatography (ERCP) findings. ( **a** ) Fluoroscopy revealing subdiaphragmatic free gas (arrow) immediately post-injection, indicative of perforation. ( **b** ) Successful deployment of an 8.5F×7 cm plastic stent for biliary drainage.

Emergency ERCP was performed. The endoscope was advanced cautiously through the cardia and pylorus into the duodenum. Following scope straightening, a guidewire was inserted into the CBD with sphincterotome assistance. The duration from endoscope insertion to successful cannulation was approximately 2.5 minutes. However, immediately after contrast injection, gas shadows were observed beneath the right hemidiaphragm, suggestive of upper gastrointestinal perforation ( Fig. 2a ). Subsequently, an 8.5F×7 cm plastic stent was deployed to ensure biliary drainage ( Fig. 2b ).

**Fig. 3:**
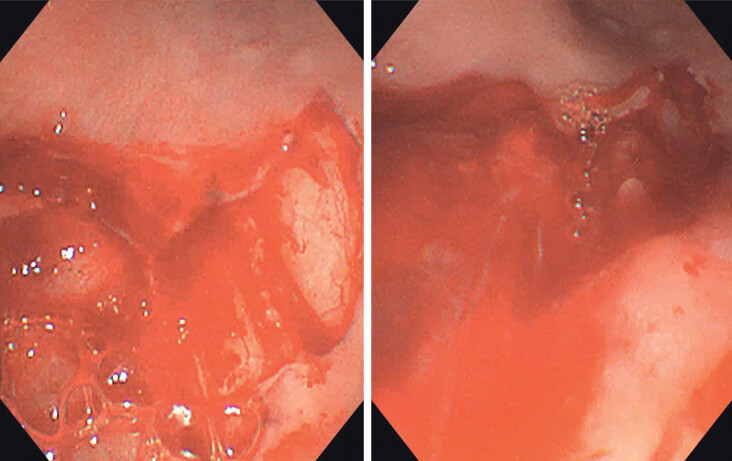
An endoscopic view upon withdrawal showing a deep longitudinal tear at the gastroesophageal junction (GEJ) with an overlying blood clot, suggestive of transmural perforation.

Systematic withdrawal of the endoscope identified a deep longitudinal tear at the GEJ, covered by a fresh blood clot, with suspected transmural involvement ( Fig. 3 ). Immediate endoscopic closure was achieved using multiple through-the-scope metallic clips ( Video 1 ). A nasogastric tube was inserted for gastric decompression, and intra-procedural contrast injection confirmed the absence of extravasation. Conservative management was initiated, comprising fasting, broad-spectrum antibiotics, proton pump inhibitors, nutritional support, and octreotide administration for mild post-ERCP hyperamylasemia.

**Video 1** Endoscopic closure of the iatrogenic gastroesophageal junction (GEJ) perforation.


**Fig. 4:**
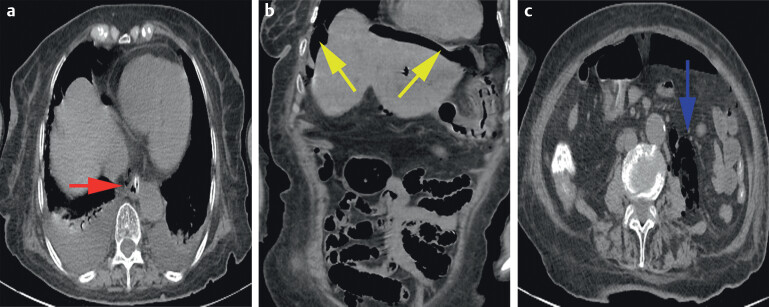
Follow-up computed tomography (CT) on day 3 demonstrating contained free gas in the periesophageal ( **a** ), perihepatic ( **b** ), and retroperitoneal regions (adjacent to the left iliac vessels) without fluid collection ( **c** ).

**Fig. 5:**
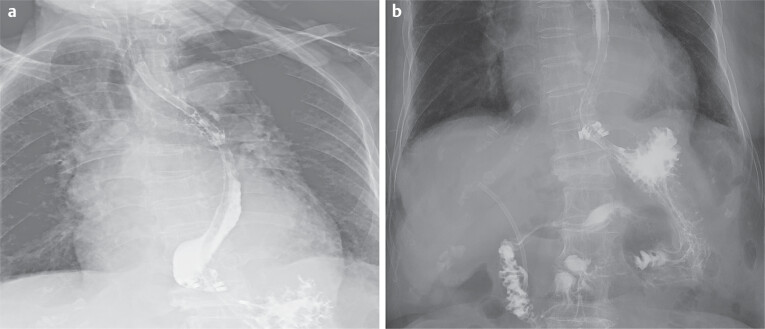
Upper gastrointestinal contrast study on day 10 showing esophageal tortuosity ( **a** ) but no contrast extravasation ( **b** ), confirming the complete healing of the perforation.

Follow-up CT on day 3 revealed multiple pockets of contained free gas in the periesophageal region, perihepatic space, and retroperitoneum adjacent to the left iliac vessels ( Fig. 4 ). An upper gastrointestinal contrast study on day 10 demonstrated esophageal tortuosity but, crucially, no contrast extravasation around the cardia, confirming complete healing and permitting diet advancement ( Fig. 5 ). By day 15, inflammatory markers and liver function tests had normalized, and the patient was discharged.

This case underscores that although GEJ perforation is rare, it may occur in elderly patients due to tissue friability and anatomical challenges. Immediate intra-procedural recognition and decisive endoscopic management are paramount for ensuring a favorable prognosis in such scenarios. 3​ ​4​

Endoscopy_UCTN_Code_CPL_1AK_2AC
